# Exploring plant responses to abiotic stress by contrasting spectral signature changes

**DOI:** 10.3389/fpls.2022.1026323

**Published:** 2023-01-20

**Authors:** Félix Estrada, Jaume Flexas, Jose Luis Araus, Freddy Mora-Poblete, Jaime Gonzalez-Talice, Dalma Castillo, Ivan A. Matus, Ana Maria Méndez-Espinoza, Miguel Garriga, Carlos Araya-Riquelme, Cyril Douthe, Benjamin Castillo, Alejandro del Pozo, Gustavo A. Lobos

**Affiliations:** ^1^ Plant Breeding and Phenomics Center, Faculty of Agricultural Sciences, University of Talca, Talca, Chile; ^2^ Instituto de Investigaciones Agropecuarias INIA-Remehue, Osorno, Chile; ^3^ Research Group on Plant Biology Under Mediterranean Conditions, Departament de Biologia, Institute of Agro-Environmental Research and Water Economy, Universitat de les Illes Balears, Illes Balears, Spain; ^4^ Department of Evolutive Biology Ecology, and Environmental Sciences, University of Barcelona, Barcelona, Spain; ^5^ Institute of Biological Sciences, University of Talca, Talca, Chile; ^6^ Departamento de Producción Forestal y Tecnología de la Madera, Facultad de Agronomía, Universidad de la República, Montevideo, Uruguay; ^7^ Instituto de Investigaciones Agropecuarias INIA-Quilamapu, Chillán, Chile; ^8^ Departamento de Producción Vegetal, Facultad de Agronomía, Universidad de Concepción, Concepción, Chile

**Keywords:** high-throughput plant phenotyping, spectroscopy, spectrometer, spectroradiometer, phenotype, physiological breeding

## Abstract

In this study, daily changes over a short period and diurnal progression of spectral reflectance at the leaf level were used to identify spring wheat genotypes (*Triticum aestivum* L.) susceptible to adverse conditions. Four genotypes were grown in pots experiments under semi-controlled conditions in Chile and Spain. Three treatments were applied: i) control (*C*), ii) water stress (*WS*), and iii) combined water and heat shock (*WS+T*). Spectral reflectance, gas exchange and chlorophyll fluorescence measurements were performed on flag leaves for three consecutive days at anthesis. High canopy temperature (***H*** *_CT_*) genotypes showed less variability in their mean spectral reflectance signature and chlorophyll fluorescence, which was related to weaker responses to environmental fluctuations. While low canopy temperature (***L*** *_CT_*) genotypes showed greater variability. The genotypes spectral signature changes, in accordance with environmental fluctuation, were associated with variations in their stomatal conductance under both stress conditions (*WS* and *WS+T*); ***L*** *_CT_* genotypes showed an anisohydric response compared that of ***H*** *_CT_*, which was isohydric. This approach could be used in breeding programs for screening a large number of genotypes through proximal or remote sensing tools and be a novel but simple way to identify groups of genotypes with contrasting performances.

## 1 Introduction

Climate change is intensifying local environmental constraints. For many areas, it is predicted that rainfall will progressively decrease, and extreme heat events will become increasingly common (Dixon et al., 2009; Rebetzke et al., 2012; Hernández-Barrera et al, 2017). In addition, the competition for water resources between human and industrial consumption will increase, affecting food security (e.g., grain production) in the coming decades (Trethowan et al., 2005; Tilman et al., 2011; Mohanty et al., 2020).

Bread wheat (*Triticum aestivum* L.) is one of the most important crops for the human diet, and one of the most widely cultivated in the world (Braun and Payne, 2013). In Mediterranean-climate areas, drought and heat have detrimental consequences during anthesis and grain filling, affecting grain yield (*GY*) and its components (Thomas and Ougham, 2014; Pinto et al., 2016; Velu et al., 2016; del Pozo et al., 2019). Breeding is one of the pillars for wheat adaptation to the threats imposed by climate change, with phenotyping being considered the bottleneck, in terms of the management of the huge information of the phenotype generated, limiting genetic advance (Araus and Kefauver, 2018; Yudina et al., 2020).

Plant phenotyping using different remote sensing approaches is receiving increasing interest among plant breeders and their users in agriculture. Among remote sensing methods, canopy, or even single leaf reflectance (represented graphically by the spectral signature) is closely related to the ability to absorb and transmit incident radiation under a particular environmental condition (Garbulsky et al., 2011), therefore a useful indicator of the plant physiological status (Peñuelas and Filella, 1998) and the Genotype by Environment (GxE) effect (Vilmus et al., 2014; Garriga et al., 2017). For instance, in a single scan, high-resolution field spectrometers acquire the reflectance of a wide range of wavelengths (350 – 2,500 nm), and this spectral signature can be related to various leaf, plant or crop traits using spectral reflectance indices (*SRIs*; relationships between particular wavelengths or spectrum bands), such as the normalized difference vegetation index (*NDVI*), which is related to leaf or canopy greenness (Babar et al, 2006a), and the photochemical reflectance index (*PRI*), associated to the xanthophyll’s activity (Gamon et al., 1992; Hernandéz-Clemente et al, 2011). Another approach to using reflectance information is through multivariate regression models to predict physiological or productive traits, that include part or the whole spectral signature (Graeff and Claupein, 2007; Sridhar et al., 2007; Winterhalter et al., 2011; Mullan, 2012; Fallon et al, 2020; Xie et al., 2020), and classification methods for direct identification of the elite genotype group (Garriga et al., 2017). Changes in the spectral signature have been associated with modifications in plant tissue properties like the hydration state or chemical composition of oak leaves (*Quercus* spp.) (Cavender-Bares et al., 2016), stomatal conductance of cotton plants (*Gossypium hirsutum*) (Vitrack-Tamam et al., 2020), or leaf photosynthetic traits (Peñuelas and Filella, 1998; Silva-Pérez et al., 2020) and carbon isotopic discrimination in the grains of wheat (Lobos et al., 2014; Garriga et al., 2021).

Nevertheless, the leafe spectral signature not only describes the biochemical an physiological state of a genotype at a certain or punctual moment, but it also integrates of environmental events throughout the growing season until the day of measurement (Marcińska et al., 2013; Lobos et al., 2019; Banerjee et al., 2020; Vitrack-Tamam et al., 2020); without ignoring the possible epigenetic effect of environmental characteristics that have impacted the seeds used for the crop (Crisp et al., 2016). Since both the uniqueness of each season’s environmental characteristics and the plasticity of the genotype are reflected in the morpho-physiological and physico-chemical traits (Elazab et al., 2012; Jäger et al., 2014; Petrov et al., 2018; Kanbar et al., 2020; Yan et al., 2020). Also, since the hydric state of the plant is closely associated with the ability to satisfy the atmospheric demand for evapotranspiration (Kudoyarova et al., 2011; Zhang et al., 2018), changes in the daily and diurnal vapor pressure deficit (*VPD*) should be accompanied by changes in the spectral signature (Magney et al., 2016). Thus, genotypes with contrasting tolerance to abiotic stresses should have different responses in their spectral signature to changing environmental conditions.

Thus, the aim of this work was to evaluate the daily changes (three consecutive days) in the leaf spectral reflectance, leaf gas exchange, chlorophyll fluorescence and pigment content, of spring wheat genotypes exposed to water stress (*WS*) and *WS* combined with heat stress (*WS+T*). It was hypothesized that genotypes more affected by environmental conditions should exhibit larger differences in spectral signature among the evaluated days. Furthermore, if the above is true, the analysis of its diurnal spectral signature should be consistent with the pattern observed in the day comparison, due to diurnal changes in air temperature and VPD.

## 2 Materials and methods

### 2.1 Selection of four genotypes for the study

Four spring wheat genotypes of contrasting canopy temperatures (*CT*) and *GY* under rainfed conditions (**Table 1**) were selected from a panel of 384 cultivars and advanced lines from CIMMYT Mexico, INIA Uruguay and INIA Chile. The panel was previously evaluated under rainfed (and high *VPD*) and irrigated (and reduced *VPD*) Mediterranean conditions, in 2011 and 2012 (del Pozo et al., 2016); the four genotypes were chosen among a subset of 104 genotypes, having a range of 80 – 83 d from sowing to earing, 4.3 – 5.6 of leaf area index at anthesis and 93 – 100 cm of plant height at anthesis.

**Table 1:** Original phenotypic characterization of the four genotypes studied (from a panel of 384 individuals under severe water deficit and high *VPD* conditions) grown under field conditions, during the 2011 and 2012 seasons.

Breeding program (origin & code)		Study Code^1^	Canopy temperature (°C)^6^		SDD^2^ (°C)	Grain yield (t ha^-1^)		YTI^3^	Δ^13^C^4^ (‰)		DE^5^ (d)	
INIA - Uruguay	Martha D16	***L_CT_-L_GY_***	18.7 ± 1.1	- 1.4 ± 0.03			1.8 ± 0.24	0.15 ± 0.05		14.7 ± 0.15		83.0
INIA - Uruguay	LE 2388	***L_CT_-H_GY_***	18.5 ± 1.4	- 1.7 ± 0.27			4.2 ± 1.01	0.49 ± 0.16		15.5 ± 0.76		79.5
CIMMYT - Mexico	Fontagro 132	***H_CT_-H_GY_***	20.4 ± 2.3	- 0.1 ± 0.67			3.9 ± 0.13	0.44 ± 0.05		14.8 ± 0.38		83.0
INIA - Chile	QUP 2569	***H_CT_-L_GY_***	19.6 ± 0.89	- 0.5 ± 0.07			2.0 ± 0.73	0.21 ± 0.06		14.4 ± 0.06		83.5
386 genotypes		minimum	18.0	-2.17			1.3	0.11		13.4		79.0
		maximum	21.6	1.37			5.5	0.71		16.1		84.0

^1^ Codification according to canopy temperature (CT; high-H and low-L) and productivity (GY; high-H and low-L).

^2^ Stress degree day (SDD) = CT- air temperature (Romero-Bravo et al., 2019).

^3^ Yield tolerance index (YTI), calculated as the relative performance of a genotype under drought with its potential yield under irrigated conditions (Ober et al., 2004; del Pozo et al., 2016).

^4^ Isotopic carbon discrimination.

^5^ Days from sowing to earing.

^6^ Measured at anthesis.

Values are means of two replicates.

### 2.2 Experimental setup

The four spring bread wheat genotypes were assessed in two controlled condition experiments, one in conventional glasshouses at the Universidad de Talca - Chile (***UTALCA*** experiment: 35°24’20” S, 71°38’5” W) in 2018, and the second in plastic growth chambers placed outdoors at the Universidad de las Islas Baleares – Spain (***UIB*** experiment: 39°38′ 17″ N, 2°38′54″ E) in 2019.

At *UTALCA*, two glasshouses were used (12 × 9 m and enclosed with alveolar polycarbonate sheets of 6 mm thickness and 86% solar transmission); one was open laterally (only the roof covered to shield plants from rain or fog), representing ambient temperature conditions (***At***), and the other was fully enclosed, and temperature controlled to ~8°C above *At* (***At+8***). At *UIB* growth chambers (1.7 × 5 m; transparent polyethylene film) were set up to provide both the *At* and *At+8* conditions. Temperature and relative humidity (*RH*) were recorded with HOBO^®^ Series H8 sensors (Onset Computer Corporation, MA, USA) at *UTALCA* and HOBO^®^ UX100-003 sensors at *UIB*. Temperature and *RH* data were used to calculate *VPD* during the experiments (**Table 2**).

**Table 2:** Environmental vapor pressure deficit (*VPD*) and air temperature during the three days of evaluations (*D1*, *D2*, and *D3*) under controlled conditions at ambient temperature (*At*; only the glasshouse roof covered) and at increased ambient temperature of 8°C (*At+8*).

Experiment	Environmental conditions	*At*				*At+8*			
		*D1*	*D2*	*D3*	Averages	*D1*	*D2*	*D3*	Averages
*UTALCA*	VPD	1.35 ± 0.57	1.50 ± 0.46	1.69 ± 0.69	1.51 ± 0.57	3.00 ± 0.64	2.97 ± 0.64	3.34 ± 0.81	3.1 ± 0.70
	(kPa)	(0.37 - 2.51)	(0.64 - 2.38)	(0.43 - 2.66)	(0.48 - 2.52)	(1.22 - 4.43)	(1.45 - 4.25)	(1.02 - 5.00)	(1.23 - 4.56)
	Air temperature	19.8 ± 4.91	21.5 ± 3.28	21.4 ± 3.28	20.9 ± 4.17	29.5 ± 3.24	29.5 ± 3.09	30.4 ± 3.41	29.8 ± 3.25
	(°C)	(8.89 - 28.1)	(14.2 - 26.6)	(11.8 - 27.0)	(11.6 - 27.2)	(19.1 - 35.3)	(21.4 - 34.7)	(18.3 - 36.6)	(19.6 - 35.5)
*UIB*	VPD	1.46 ± 0.33	1.42 ± 0.30	1.59 ± 0.31	1.49 ± 0.31	3.61 ± 1.53	3.61 ± 1.25	3.66 ± 1.09	3.62 ± 1.29
	(kPa)	(1.02 - 2.32)	(0.91 - 2.00)	(0.61 - 2.24)	(0.85 - 2.19)	(0.72 - 7.42)	(0.76 - 6.00)	(0.60 - 6.09)	(0.69 - 6.50)
	Air temperature	26.2 ± 2.12	26.0 ± 1.66	26.0 ± 1.07	26.1 ± 1.62	34.2 ± 5.51	34.5 ± 4.33	35.6 ± 3.34	34.8 ± 4.39
	(°C)	(23.0 - 29.9)	(22.7 - 29.2)	(23.4 - 27.7)	(23.0 - 28.9)	(22.2 - 43.1)	(23.6 - 41.6)	(23.4 - 41.8)	(23.1 - 42.2)

In the *UTALCA* (Chile) and *UIB* (Spain) experiments, values represent the average between 9:30 and 16:30 h ± standard deviation, with the recorded ranges between brackets.

Genotypes were exposed to the following growing conditions during anthesis: i) control (***C***: substrate ~75% pot water capacity and ambient temperature); water stress (***WS***: under *At*, substrate ~30 and 50% of pot water capacity at *UTALCA* and *UIB* experiments, respectively); and iii) water and heat shock combined (***WS+T***: water deficit and ambient temperature increased by around 8°C).

For the *UTALCA* experiment, 40 seeds of each genotype were sown in 20 L plastic containers (radius: 15 cm; height: 50 cm), filled with a 6:1:1 mixture of river sand, organic soil and perlite. For the *UIB* experiment, 20 seeds of each genotype were sown in 10 L plastic containers (radius: 12.5 cm; height: 19.5 cm), filled with a 1:1 mixture of river sand and perlite. In both experiments, the seeds were distributed in concentric circles, sowing an outer ring to minimize edge effects at the root level (i.e., plants growing in contact with the wall of the pot; 10 and 5 seeds in *UTALCA* and *UIB*, respectively) and the inner rings with seeds of plants that were to be measured (30 and 15 seeds in *UTALCA* and *UIB*, respectively). Both in *UTALCA* and *UIB*, fertilizer was applied weekly, comprising 1,000 ml of full-strength Hoagland’s nutrient solution (Hoagland and Arnon, 1950).

### 2.3 Experimental design and measurements

The two trials were conducted as a completely randomized design, with three replicates (pots) per treatment and genotype. To determine the maximum water holding capacity, each pot was watered until saturation and weighed after 24 h of drainage to determine the amount of water required to reach 100% pot capacity. During the experiments, pots were weighed daily. All pots were treated as control plants (*C*) until flag leaf blades were fully unfolded (Z41; Zadoks et al., 1974). From Z41 to anthesis (Z68), plants of each genotype were divided in two groups: 1/3 were kept as *C* and 2/3 were exposed to *WS* conditions. At Z68, plants under *WS* were again divided in two groups, half continued in *WS* while the other plants were exposed to *WS+T* in the *At+8* environment.

The leaf spectral signature and gas exchange was determined for each genotype and treatment. In the *UTALCA* and *UIB* experiments, once the combined stress (*WS+T*) was imposed, measurements were performed immediately on three consecutive days (*D1*, *D2*, and *D3*), at solar zenith, just before the plateau of the maximum daily atmospheric demand for water. In addition, to test whether genotypic differences within the day (diurnal progression) were consistent with those in reflectance observed between days, on *D3* the spectral reflectance was evaluated at 10:00, 11:30, 13:00, 14:30, and 16:30 h (UTC - 4), in the *UTALCA* experiment. Spectral reflectance and gas exchange evaluations were carried out on the middle third of healthy and sun-exposed flag leaves. At *UTALCA*, three flag leaves per pot were selected for each assessment, while at *UIB*, two flag leaves were considered per replicate. Different leaves were considered in each evaluation for both progression between days and within the day.

#### 2.3.1 Spectral reflectance

At *UTALCA*, the reflectance was recorded with a FieldSpec 3 Jr. portable spectrometer (Analytical Spectral Devices ASD Inc., Boulder, CO, USA) over a spectral range 350 – 2,500 nm, and a spectral resolution of 3 nm from 350 to 1,000 nm and 30 nm from 1,001 to 2,500 nm. The fiber was inserted into a contact probe device (ASD Inc., Boulder, CO, USA) equipped with a halogen light (5 W), generating a measuring spot of ~5 mm radius. The spectrometer was calibrated every 15 min with a white reference tile (Spectralon^®^, ASD Inc., Boulder, CO, USA). The equipment was configured to integrate three samples per scan, and ten scans per leaf (as described in Lobos and Poblete-Echeverría, 2017). The data were extracted using View Spec Pro 2008 software (ASD Inc., Boulder, CO, USA). The exploratory analysis of the spectral reflectance data was performed using SK-UTALCA software (Lobos and Poblete-Echeverría, 2017).

At *UIB*, reflectance measurements were performed with a Vis/NIR optical spectrometer (Jaz-EL350, Ocean optics, Dunedin, FL, USA) equipped with a halogen tungsten light source (4.75 W) and a QP600-1-SR-BX optical fiber (Ocean Optics, Inc., Dunedin, FL, USA; spectral range 400 – 900 nm), with 0.3 nm spectral resolution. The calibration parameters were adjusted to 10 ms for the integration time, with a light intensity of 2,500 (counts). Before measurement, the spectrometer was calibrated to 100% reflectance using a white reference panel (Ocean Optics, Dunedin, FL, USA), while the dark calibration was obtained with a black panel. The equipment was configured to integrate ten scans per leaf. Because there was not enough space to take measurements in the growth chambers, spectral reflectance was measured in a room (25°C) adjacent to the chambers.

#### 2.3.2 Leaf gas exchange, chlorophyll fluorescence and pigment content

At *UTALCA*, leaf net CO_2_ assimilation (*An*), the stomatal conductance (*gs*) and the leaf temperature (*T_l_*) were evaluated using a CIRAS 2 infrared gas analyzer (IRGA) (PP Systems, Amesbury, MA, USA), with a narrow-leaf cuvette (1.7 cm^2^) at a flow rate of 250 ml min^-1^, CO_2_ concentration of 400 ppm, cuvette temperature of 25°C, and photosynthetically active radiation (*PAR*) of 1,500 µmol m^-2^ s^-1^. At *UIB*, measurements were performed with a Li-6400xt portable photosynthesis system (LI-COR Inc., Lincoln, Nebraska, USA). The environmental parameters of the leaf chamber were adjusted to equal those of the *UTALCA* experiment, except for the temperature of the cuvette, which was set at room temperature.

In *UTALCA* experiment, chlorophyll fluorescence (*Chl_f_*) was assessed using a portable pulse amplitude modulated fluorometer (PAM-2500, Walz, Germany). The minimum and the maximum yield of fluorescence under dark conditions (*Fo* and *Fm*, respectively), were measured on leaves adapted to darkness for 20 minutes with a Leaf-Clip (Leaf-Clip Holder 2030- B, Walz, Germany). Then, a rapid light curve (*RLC*) was performed in the same leaf spot. For that, the equipment was programmed to emit 10 pulses of actinic light at different levels of photosynthetically active radiation (*PAR*), which increased from 0 to 1,982 μmol m^-2^ s^-1^ (the time between each *PAR* level was six seconds). At each *PAR*, the minimum calculated and the maximum yield of fluorescence under light conditions (~*Fo´* and *Fm´*, respectively), were automatically recorded.

Anthocyanin content was estimated using a non-destructive portable chlorophyll meter (Dualex, Force A, France); values are given in relative absorbance units from 0 to 1.5.

### 2.4 Data analyses

The genotypic (*G*) variability of the spectral signature under each treatment (environment-*E*) was assessed through direct comparison (ANOVA and *p-values < 0.05*) of the spectral signatures at the wavelength level (Lobos et al., 2019). Thus, the first reflectance measurements (i.e., measurements of *D1* compared with *D2* and *D3* in case of the daily analysis, and measurement at 10:00 h with each moment of measurement in the diurnal progression analysis) were compared (ANOVA, *p-values < 0.05*; n = 9 and 6 in *UTALCA* and *UIB* experiments, respectively) to the subsequent ones. When significant differences were found (Tukey’s multiple comparison test, *p-values < 0.05*) between the said spectral signatures, the percentage change with respect to the first measurement was calculated and plotted.

To establish statistical differences in the gas exchange performance of the genotypes and environments, multifactor ANOVA (*p-values < 0.05*; n = 9 and 6 in *UTALCA* and *UIB* experiments, respectively) was carried out on *T_l_*, *gs*, and *An*. When significant differences were found, Tukey’s test (*p-values < 0.05*) was applied. To identify the genotypic variability of biochemical limitations of photosynthesis to the generated environmental conditions, the relationship between *An* and *gs* was studied through regression analysis (Medrano et al., 2002; Gago et al., 2020).

Finally, in the *UTALCA* experiment, the spectral reflectance was also used to calculate the normalized difference vegetation index (*NDVI*; [R780 - R670]/[R780 + R670], Babar et al., 2006a) and the photochemical index (*PRI*; [R570 - R531 - R670]/[R570 + R531 + R670], Hernández-Clemente et al., 2011). Pearson correlation analyses were performed using both SRI and vapor pressure deficit (*VPD*), relative humidity (*RH*), air temperature (*T_a_*), anthocyanin content (*Anth*) and leaf temperature (*T_l_*). These *SRIs* were also used to characterize the behavior of the genotypes, both between and within the day.

Statistical analysis was performed with RStudio v. 1.2.1335 (RStudio Inc.).

## 3 Results

### 3.1 Environmental conditions during the experiment

The average temperature and *VPD* was increased by 8°C and 1.6-2.3 kPa, respectively, at the elevated temperature regime, in both experiments (**Table 2**). However, temperature at *At* regime – and, consequently, at all other regimes – was higher in the *UIB* experiment compared to the *UTALCA* experiment. During *D3* of the diurnal progression study (*UTALCA*), the ambient temperature and *VPD* gradient also increased from 09:00 h (*At*: 14.2°C and 0.65 kPa; *At+*8: 25.0°C and 2.85 kPa) to a peak between 14:00 and 16:00 h (*At*: 25.9°C and 2.41 kPa; *At+8*: 33.2°C and 4.1 kPa).

### 3.2 Determining spectral signature stability throughout daily progression analysis

**Figure 1:**
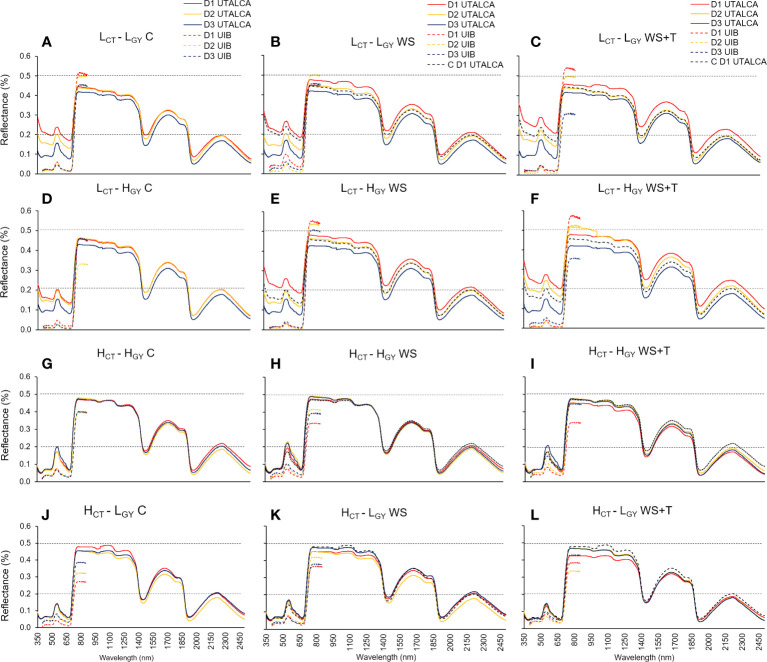
Leaf spectral signatures assessed on three days (*D1*; red line, *D2*; yellow line; and *D3*; blue line) in four spring bread wheat genotypes, growing under control conditions (*C*, substrate ~75% pot water capacity and ambient temperature) **(A, D, G, J)**, soil water stress (*WS*, substrate of ~30 and 50% of the pot water capacity in the *UTALCA* and *UIB* experiments, respectively; ambient temperature) **(B, E, H, K)**, and combined soil water and heat stress conditions (*WS+T*, substrate of ~30 and 50% of the pot water capacity in the *UTALCA* and *UIB* experiments, respectively; ambient temperature increased by around 5 – 7°C) **(C, F, I, L)**. According to the canopy temperature (*CT*; high-*H* and low-*L*) and productivity (*GY*; high-*H* and low*-L*), genotypes were designated as: *L_CT_-L_GY_*, *L_CT_-H_GY_*, *H_CT_-H_GY_*, and *H_CT_-L_GY_*. Measurements were performed just before the plateau of the maximum daily atmospheric demand for water (13:00 h). Data from the *UIB* experiment ranges from 400 to 900 nm. For reference purposes only, the horizontal black dashed lines represent 20% and 50% of reflectance; the dashed red line on the *WS* and *WS+T* curves represents the *D1* leaf spectral signature in *C*; n = 9 in the *UTALCA* experiment and n = 6 in the *UIB* experiment.

**Figure 2:**
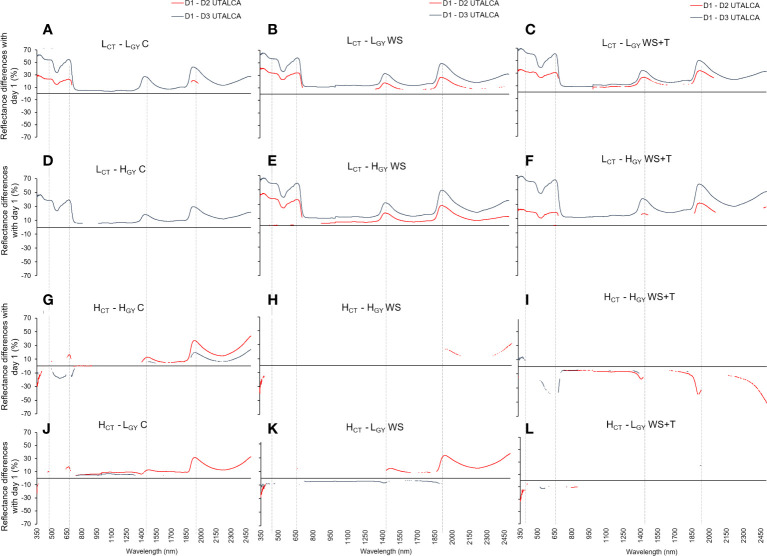
Statistical differences in spectral reflectance calculated as the percentage change in the wavelength level between days of assessment (*D1* to *D3: D1*-*D2* red line, and *D1*-*D3* blue line; at each wavelength, interruptions in the lines indicate sections without statistical differences, p < 0.05) in four spring bread wheat. Genotypes and treatments are indicated in **Figure 1**. Measurements were performed just before the plateau of the maximum daily atmospheric demand for water (~13:00 h). n = 9. The dashed vertical lines indicate the peaks around 380, 680, 1450, and 1950 nm.

The spectral reflectance signature of plants growing under *C, WS*, and *WS+T* treatments over time (*D1* to *D3*) varied among genotypes according to the *CT* performance (**Figure 1**). The spectral reflectance of low *CT* genotypes (***L_CT_-H_GY_*** and ***L*** *_CT_-* ***L*** *_GY_*) showed greater variation between days (**Figures 1A–F**) than did that of high *CT* genotypes (***H_CT_-L_GY_*** and ***H_CT_-H_GY_***) (**Figures 1G–L**). Although somewhat less evident than under stress conditions, this behavior was also reflected in the control condition. The major changes in high *CT* genotypes were observed on *D2* (red lines in **Figures 2G, J**), while the greatest changes were found in the low *CT* genotypes in *D3* (blue lines in **Figures 2A, D**). In addition, genotypes showed different patterns throughout the day according to their *CT*. The mean reflectance signatures of low *CT* genotypes continued to decrease from *D2* to *D3*, with respect to *D1* (**Figures 2A–F**), while in high *CT* genotypes, reflectance stopped changing or increased in the direction of their initial condition in *D1* (**Figures 2G–L**). The effects of *WS* and *WS+T* treatments on the spectral signature performance was lower in low *CT* genotypes compared to high *CT* genotypes, particularly in the *UTALCA* experiment (**Figure 2**). In the case of ***H*** *_CT_*-***H*** *_GY_*, greater differences were observed in the patterns of the spectral signature under *WS* and *WS+T*. Under *WS* treatment (**Figure 2H**), the reflectance in the *VIS* and the first part of the *NIR* increased during *D2* and *D3* with respect to *D1* (negative differences), but at wavelength > 1,400 nm, the reflectance decreased compared to *D1* (positive values). While under *WS+T* conditions, in almost the entire spectra, the reflectance during *D2* and *D3* increases, compared to *D1* (**Figure 2J**). In the case of ***H*** *_CT_*-***L*** *_GY_* under *WS*, the spectral reflectance was reduced on *D2*, compared to *D1*, but the opposite was observed in *D3* (**Figure 2K**). The same tendency was observed for the *WS+T* treatment.

Unlike in the *UTALCA* experiment, where the differences between the reflectance were detected from 350 nm onwards, especially in low *CT* genotypes, in the *UIB* experiment, the divergences were evident at 700 – 900 nm. However, in both experiments the electromagnetic spectrum regions with the greatest percentage of change in relation to *D1* were 350 – 740 nm (high *CT*: -33 – 15%; low *CT*: 2 – 70%), 1350 – 1600 nm (low *CT*: 8 – 36%; high *CT*: -17 – 12%), and 1850 – 2500 nm (low *CT*: 0 - 47%; high *CT*: -12 – 43%) (**Figure 2**). There were four peaks that differed in intensity between *CT* groups: 380, 680, 1,450, and 1,950 nm.

### 3.3 Determining spectral signature stability through diurnal progression analysis

**Figure 3:**
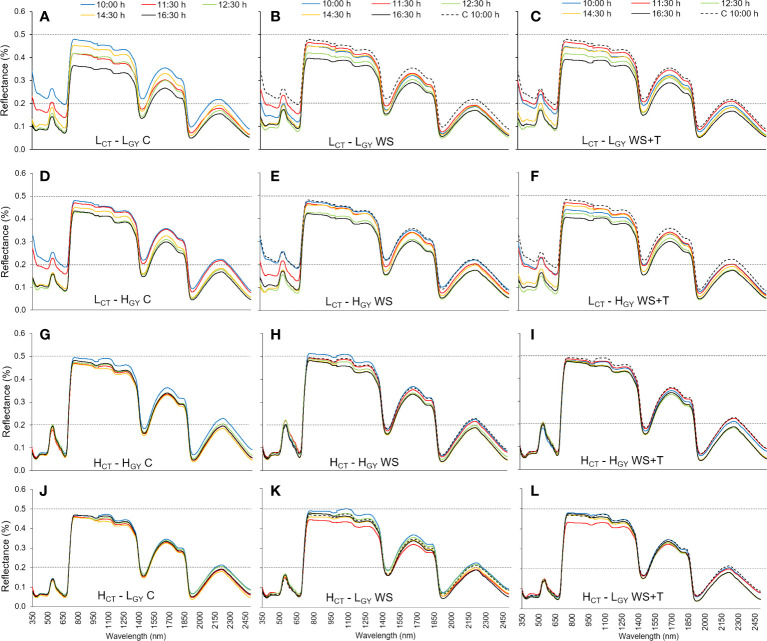
Leaf spectral signatures throughout the third day of evaluation (10:00 h: blue line, 11:30 h: red line, 12:30 h: green line, 14:30 h: orange line, and 16:30 h: black line) of four spring bread wheat genotypes. Genotypes and treatments are indicated in **Figure 1**. For reference purposes only, the horizontal black dashed lines represent 20% and 50% reflectance; the 10:00 h *C* measurements of each genotype are represented as dashed blue lines on the *WS* and *WS+T* signatures; n = 9.

**Figure 4:**
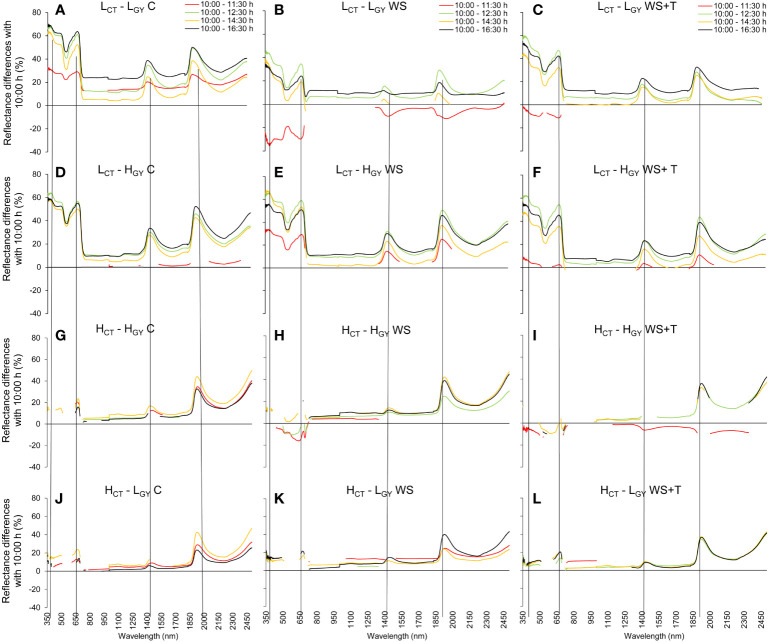
Statistical differences in the spectral reflectance calculated as the percentage change in wavelength level during the day (10:00/11:30 h red line, 10:00/12:30 h green line, 10:00/14:30 h yellow line, and 10:00/16:30 h black line; at each wavelength, interruptions in the lines indicate sections without statistical differences, p < 0.05) of four spring bread wheat genotypes. Genotypes and treatments are indicated in **Figure 1**. n = 9. The dashed vertical lines indicate the peaks around 380, 680, 1450, and 1950 nm.

Spectral signature changes recorded within the day (10:00 – 16:30 h) (**Figures 3**, **4**) followed similar patterns to those described for the daily progression analysis. Low *CT* genotypes, also showed greater differences than high *CT* genotypes, when comparing the changes in spectral signatures recorded at 10:00 with those recorded later in the afternoon. The spectral signature performance at 12:30 and 16:30 h appears to be the same in the high *CT* genotypes (**Figures 4H, I, K, L**); also, under the control conditions in the four genotypes (**Figures 4A, D, G, J**).

### 3.4 Gas exchange assessments

**Figure 5:**
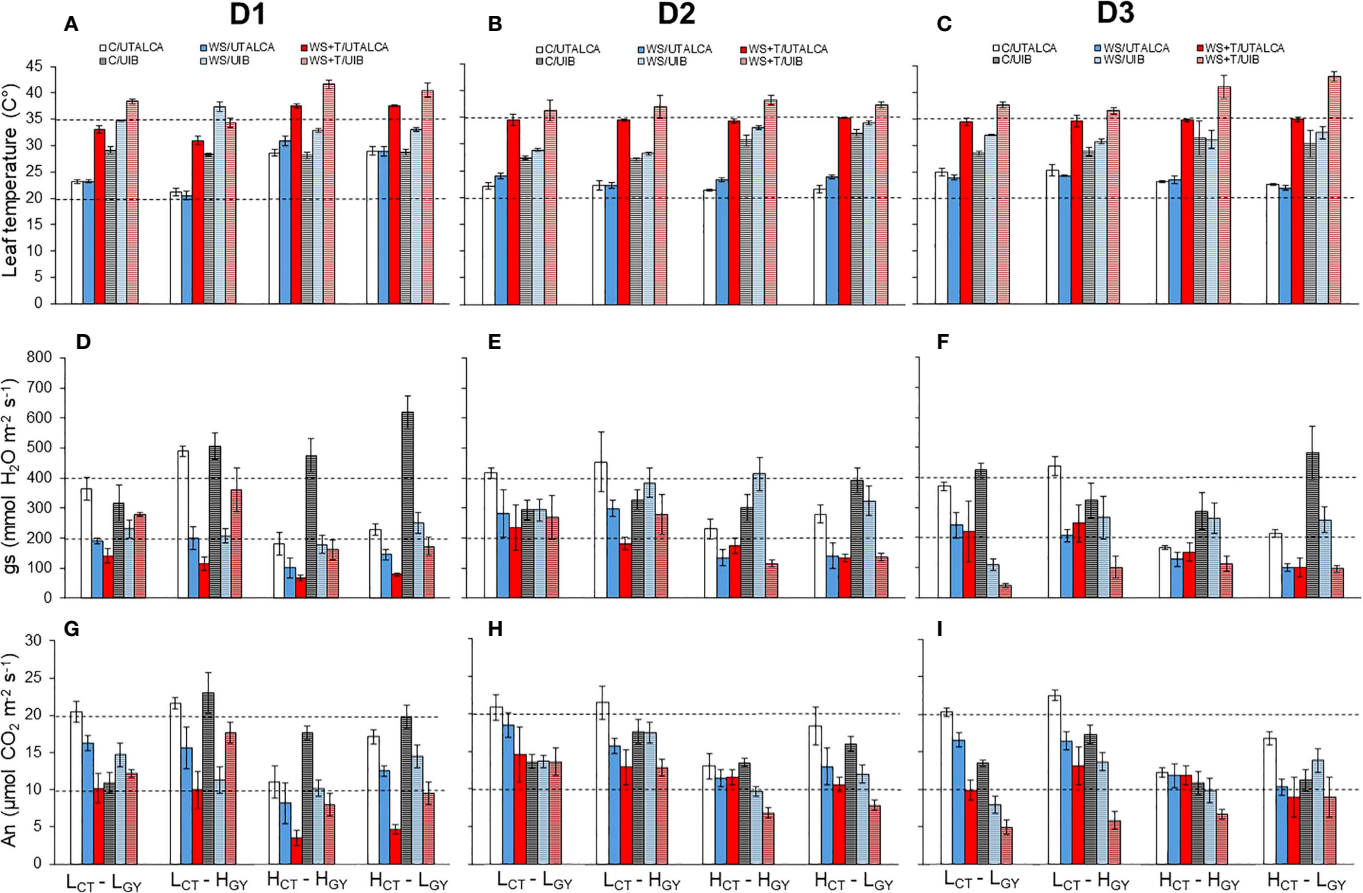
Leaf temperature (*T_l_*) **(A–C)**, stomatal conductance (gs) **(D–F)**, and leaf net CO2 assimilation (An) **(G–I)** of four genotypes evaluated at three days: *D1* **(A, D, G)**, *D2* **(B, E, H)**, and *D3* **(C, F, I)**. Genotypes and treatments are indicated in **Figure 1**. Fully shaded columns represent data collected in the *UTALCA* experiment, while partly shaded columns are data collected in the *UIB* experiment. The genotypes in each figure are ordered from highest to lowest *gs* according to *C* in the *UTALCA* experiment. Values are averages ± standard error (n = 3).

Regarding the initial selection of the genotypes by leaf temperature (**Table 1**), the results show that these *CT* characteristics were maintained in the present study when analyzing the gas exchange performance of the genotypes (**Figures 5A–C**). The ANOVA performed for each day of evaluation, at *UTALCA* experiment, shows statistical differences between genotypes in *gs*, *An*, *Fm*, *Fm´*, *Fo* and *~Fo´* during the three days, meanwhile *T_l_* just on D3. In the case of the treatments, each day shows significant differences among them. For the interaction between genotype and treatment, we found significant differences for the *gs* and the fluorescence variables (**Supplementary Table 2**). In the case of the *UIB* experiment, the genotypes showed statistical differences in *An* on the three days. During *D2*, both low *CT* genotypes showed lower *T_l_* (4°C minus) than high *CT* genotypes. in terms of the treatments, similar to *UTALCA*, showed statistical differences at each day. In *D1*, the three variables have significative interactions (**Supplementary Table 2**). In general terms, the high *CT* genotypes had lower *gs* and *An* values across both experiments (**Figures 5D–I**), than the low *CT*. In consequence, the leaf temperature of both high *CT* genotypes was higher compared to low *CT* genotypes. In all genotypes, *gs* and *An* were strongly reduced under *WS* and *WS+T* (**Figures 5 D–I**). The two genotypes with lower CT tend to had higher fluorescence level during *D1* and *D3*. Genotypes under *C* conditions in the *UTALCA* experiment showed similar *gs* and *An* levels during the three days of the experiments (**Figures 5D–I**).

The high *CT* genotypes were the most affected by the combined stress conditions on *D1*, however *gs* and *An* progressively reached the same values of *WS* conditions on *D3* (**Figures 5E, F, H, I**). In both trials, ***H_CT_-H_GY_*** show the greater capacity to overcome the *WS+T* condition than ***H_CT_-L_GY_*,** which in the case of the *UTALCA* experiment achieves almost similar *gs* values than under *C*.

The relationship between *An* and *gs* indicated that for a given value of *gs*, *An* was higher in the *UTALCA* experiment, in all genotypes (**Figure S3**). Although with different IRGAs, it is interesting to note that at the same *gs* level, the differences in *An* between experiments were lesser in the high-yielding genotypes (**Figures S3A, C**) than in the low-yielding genotypes (***L_CT_-L_GY_*** and ***H_CT_-L_GY_***) (**Figures S3B, D**). Greater genotypic differences were found at values of *gs* above 150 mmol H_2_O m^-2^ s^-1^ in the *UTALCA* experiment, while at *UIB*, genotypic variability was evident at lower *gs* levels (**Figures. S3E, F**). In these sense, two groups were identified according to *CT* (i.e., Lower and High). Despite the dissimilarities associated with experiment location, the ***H_CT_-H_GY_*** genotype always had a lower ratio *An/gs* (**Figures S3E, F**).

### 3.5 Relationship between spectral reflectance indices with environmental and physiological variables

The results of the correlation analysis between the studied *SRIs* and both the environmental (*VPD*, *HR*, air temperature) and foliar (temperature and anthocyanins) characteristics varied according to the index (**Table 3**). Thus, at compare *NDVI* and *PRI*, the first one showed a wide and significance association in ***L*** *_CT_*-***L*** *_GY_* and ***L*** *_CT_*-***H*** *_GY_*. On the other hand, correlations with the same genotypes showed higher variability with *PRI*.

**Table 3:** Pearson correlation analyses (*r* and *p-values*) of the relationships between spectral reflectance indexes (normalized difference vegetation index; *NDVI* and photochemical index; *PRI*), with the vapor pressure deficit (*VPD*), relative humidity (*RH*), air temperature (*T_a_*), anthocyanins content (*Anth*), and leaf temperature (*T_l_*) in the *UTALCA* experiment during the 2018 season, for four spring bread wheat genotypes on three days of evaluation for plants growing under control conditions (*C*), soil water stress (*WS*) and combined soil water and heat stress conditions (*WS+T*).

Genotype	Treatments & Statisitics		NDVI *vs.*					PRI *vs.*				
			VPD (kPa)	RH (%)	T_a_ (°C)	Anth. (index)	T_l_ (°C)	VPD (kPa)	RH (%)	T_a_ (°C)	Anth. (index)	T_l_ (°C)
***L_CT_-L_GY_***	C	r	0.82	-0.86	0.77	0.75	0.67	0.74	-0.83	0.67	0.65	0.66
		*P value*	***0.007***	***0.003***	***0.016***	***0.021***	***0.048***	***0.022***	***0.006***	***0.048***	***0.060***	***0.055***
	WS	r	0.86	-0.55	0.79	0.74	0.41	0.69	-0.64	0.53	0.59	0.30
		*P value*	***0.003***	*0.125*	***0.011***	***0.022***	*0.271*	***0.039***	*0.061*	*0.143*	*0.091*	*0.427*
	WS+T	r	0.23	0.79	0.47	0.81	0.63	0.20	0.73	0.43	0.78	0.54
		*P value*	*0.547*	***0.012***	*0.197*	***0.008***	*0.071*	*0.608*	***0.024***	*0.252*	***0.013***	*0.132*
***L_CT_-H_GY_***	C	r	0.70	-0.62	0.55	0.78	0.48	0.67	-0.64	0.49	0.76	0.42
		*P value*	***0.035***	*0.072*	*0.127*	***0.014***	*0.189*	***0.050***	*0.066*	*0.177*	***0.017***	*0.255*
	WS	r	0.75	-0.78	0.73	0.41	0.80	0.55	-0.59	0.55	0.58	0.91
		*P value*	***0.0202***	***0.012***	***0.025***	*0.275*	***0.009***	*0.126*	*0.093*	*0.123*	*0.101*	***0.001***
	WS+T	r	0.11	0.81	0.29	0.86	0.73	-0.02	0.83	0.15	0.80	0.65
		*P value*	*0.778*	***0.008***	*0.455*	***0.003***	***0.025***	*0.961*	***0.006***	*0.694*	***0.010***	*0.061*
***H_CT_-H_GY_***	C	r	-0.72	0.54	-0.46	0.11	-0.39	-0.13	-0.46	-0.61	0.83	-0.81
		*P value*	***0.028***	*0.137*	*0.215*	*0.785*	*0.298*	*0.730*	*0.208*	*0.080*	***0.006***	***0.009***
	WS	r	0.38	-0.65	0.30	-0.41	0.23	-0.54	0.63	-0.51	0.46	-0.40
		*P value*	*0.314*	*0.058*	*0.433*	*0.270*	*0.554*	*0.132*	*0.066*	*0.165*	*0.216*	*0.283*
	WS+T	r	-0.01	0.28	0.09	-0.26	0.31	0.29	-0.75	0.05	0.38	-0.31
		*P value*	*0.972*	*0.463*	*0.822*	*0.492*	*0.419*	*0.449*	***0.020***	*0.895*	*0.313*	*0.423*
***H_CT_-L_GY_***	C	r	-0.03	0.41	0.13	-0.04	0.10	-0.36	0.22	-0.31	0.41	-0.40
		*P value*	*0.945*	*0.271*	*0.737*	*0.928*	*0.795*	*0.347*	*0.570*	*0.420*	*0.279*	*0.288*
	WS	r	-0.55	0.39	-0.38	0.20	-0.06	-0.82	0.57	-0.57	0.08	-0.05
		*P value*	*0.121*	*0.296*	*0.310*	*0.602*	*0.885*	***0.007***	*0.111*	*0.112*	*0.839*	*0.899*
	WS+T	r	-0.58	0.18	-0.63	0.63	-0.65	-0.58	-0.06	-0.67	0.87	-0.83
		*P value*	*0.102*	*0.644*	*0.072*	*0.070*	*0.057*	*0.101*	*0.882*	***0.047***	***0.003***	***0.005***

According to the canopy temperature (*CT*; high-*H* and low-*L*) and productivity (*GY*; high-*H* and low-*L*) genotypes were designated as: ***L*** *_CT_-* ***L*** *_GY_*, ***L*** *_CT_-* ***H*** *_GY_*, ***H*** *_CT_-* ***H*** *_GY_*, and ***H*** *_CT_-* ***L*** *_GY_*. Measurements were performed just before the plateau of the maximum daily atmospheric demand for water (~13:00 h); n = 9. significative p-values are denoted in bold.

### 3.6 Consistency between reflectance and fluorescence assessments

**Figure 6:**
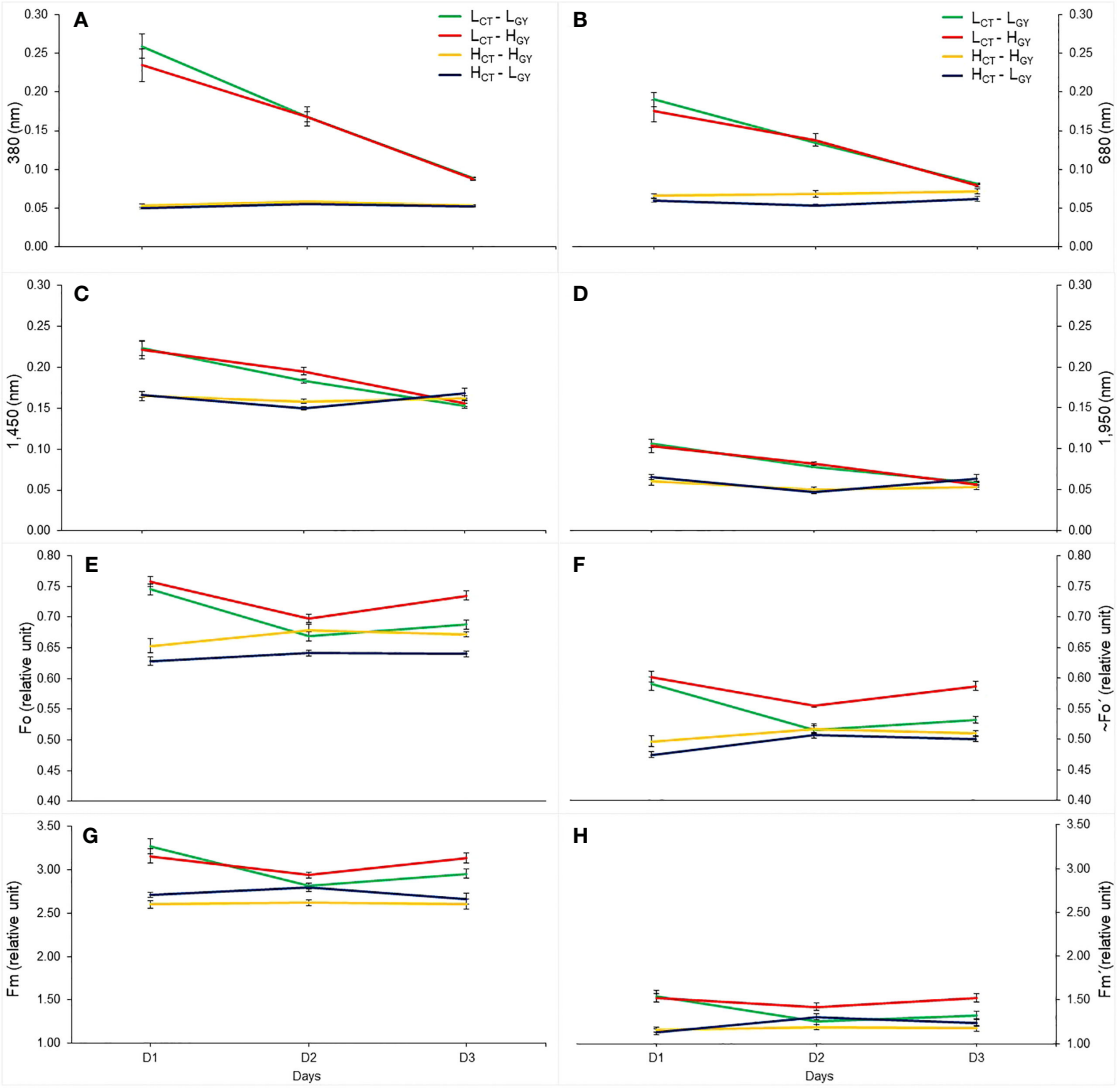
The mean value, considering the three treatments, of the selected wavelength reflectance (380 nm; **(A)**, 680 nm; **(B)**, 1,450 nm; **(C)**, 1,950 nm; **(D)**, and chlorophyll fluorescence variables (*Fo*; **E**, *~Fo´*; **F**, *Fm*; **G**, *Fm´*; **H**), at *UTALCA* experiment, measured in each genotype (*L_CT_-L_GY_*; green, *L_CT_-H_GY_*, red; *H_CT_-H_GY_*; orange, *H_CT_-L_GY_*, blue), on the three days of evaluations (*D1*, *D2*, and *D3*). Values are averages ± standard error per day (n = 9).

A similar trend of changes was observed when comparing daily mean values of reflectance at 380, 680, 1,450 and 1,950 nm (where differences were most evident when comparing *D1* versus *D2* and *D3*), with daily mean values of chlorophyll fluorescence variables. In other words, the low *TC* genotypes showed greater variability at wavelengths 380, 680, 1,450, 1,950 nm and in *Fo*, *~Fo’*, *Fm* and *Fm’*, from *D1* to *D3* (**Figures 6A–D**), whereas in the high *TC* genotypes smaller changes were observed between days. In fact, ***H*** *_CT_-* ***H*** *_GY_* had no change in *Fm* and *Fm’* between days, whereas ***H*** *_CT_-* ***L*** *_GY_* had the same performance in *Fo* and *~Fo’* (**Figures 6E–H**). Also, higher and significative correlations of gas exchange (*T_l_*, *gs* and *An*) and fluorescence variables with the wavelength’s reflectance (at 380, 680, 1,450 and 1,950 nm) were found (**Supplementary Table 1**). During *D1*, the gas exchange and fluorescence variables have higher correlations with the reflectance of the wavelengths especially under *WS* and *WS+T*. The physiological traits correlate better with the 380 and 680 nm; *gs* and *An*, correlate better with 380 nm, while fluorescence variables with the four wavelengths.

## 4 Discussion

The environmental data (**Table 2**), and specifically the range of ambient temperatures and *VPD*, suggest that plants at *UTALCA* under *WS+T* were subjected to a moderate atmospheric demand (i.e., 1.5 KPa) and moderate heat stress (i.e., 32°C) (Lizana and Calderini, 2013). It has been reported that gas exchange, specifically *gs*, is reduced by around 17% in anthesis when plants are subject to an environment with heat shock of 32°C and full irrigation (Djanaguiraman et al., 2020). The results of this study showed a reduction of *gs* and *An* higher than 30% due to the plants being subjected to two stressor factors (water and heat shock). In the *UIB* experiment, in addition to higher *VPD* (> 3.5 KPa) in *WS+T* condition, the high night temperatures (night mean of 24°C at *UIB*, versus 13°C at *UTALCA*, data not shown) were also involved in the plant response to stress environment (i.e., spectral reflectance and gas exchange variables) (Fleitas et al., 2020; Fakhet et al., 2021). Furthermore, plants were exposed to severe environmental stress than in the *UTALCA* experiment.

Different studies report an anisohydric and isohydric performance of wheat genotypes, although early works classified mainly as anisohydric species (Henson et al., 1989; Tardieu and Simonneau, 1998), both types of stomatal control have been described in wheat genotypes. The anisohydric stomatal response is related to a less conservative water strategy in terms of transpiration (De Boeck et al., 2011; De Boeck et al., 2016). Genotypes with this type of response have higher rates of transpiration and *gs*, until drought becomes severe and stomatal closure occurs (Gallé et al., 2013; Liu et al., 2018). Different mechanisms are associated with this response such as ABA synthesis at root level (Gallé et al., 2013; Saradadevi et al., 2014; Saradadevi et al., 2016; Giusti et al., 2017), osmotic adjustment, the increase of osmoprotective compounds (Smirnov et al., 2020) and the increase of antioxidant capacity (Gallé et al., 2008) at leaf level (Smirnov et al., 2020). On the contrary, the isohydric stomatal response has the opposite behavior, more conservative water losses by faster stomatal closure under water stress conditions, due to a higher sensitivity to abscisic acid signals (Blum, 2015). In this study, low *CT* genotypes maintained higher *gs* levels in both stress environments, representing a nearly anisohydric stomatal response (**Supplementary Figures S4E, F**). On the other hand, the high *CT* genotypes in the *WS+T* condition, showed an isohydric stomatal regulation, based on a stronger reduction of *gs* and *An* at *D1*, reflecting a greater sensitivity to abscisic acid signals (Blum, 2015). With such stomatal control the dispersion of *gs* and *An* across days remains low and constant (**Figures S4E, F**); a more conservative strategy to maintain tissues water status, at the cost of low CO_2_ assimilation due to stomatal closure (Davies and Pereira, 1992; Maroco et al., 1997; Negin and Moshelion, 2017; Fallon et al., 2020). As in this study, Bayoumi et al. (2015) compared the plant *CT* of wheat genotypes at zenith, and were able to recognize the most contrasting isohydric and anisohydric material.

Spectral reflectance performance can be related to anisohydric or isohydric performance. For example, measuring at zenith the *PRI* and *NDVI*, Vaz et al. (2016) was able to differentiate between anisohydric and isohydric grapevines cultivars under drought conditions. As in this work, isohydric cultivar did not showed significant variations in their spectral reflectance signatures or in the *NDVI* and *PRI* pattern compared to the anisohydric cultivar. Similar results were reported by Sobejano-Paz et al. (2020) comparing species with different stomatal control under water deficit; maize (anisohydric) and soybean (isohydric).

In particular, the changes in the *PRI* are associated with xanthophyll activity which increase under stress (Gamon et al., 1992). Hence, the change of *PRI* reflects the variation of photosynthetic activity in a certain period (i.e., through the days and hours), as a response to the stomatal and xanthophylls activity under different environments (Gerhards et al., 2018). Likewise, changes in *NDVI* could be related to tissue hydric status, the photosynthetic activity considering the total canopy green area, or at the leaves level (chlorophylls content) (Gamon et al., 2015; Duan et al., 2017) and also, by stomatal control. In the present work, the low *CT* genotypes presented higher correlations of *SRIs* (*NDVI* higher than *PRI*) with both the anthocyanin content and the recorded environmental variables, compared to high *CT* genotypes (**Table 3**); nevertheless, the interpretation of *NDVI* is far from easy, and changes in the *NDVI* could occur by mechanisms other than chlorophylls loss (Atherton et al., 2020).

In addition, the daily behavior of the spectral signature (**Figure 2**) and the calculated *SRIs* (**Supplementary Figure S1**) were consistent with the pattern during the day (**Figure 4** and **Supplementary Figure S2**); in all tested environmental conditions, increase in *VPD* and environmental temperature throughout the day resulted in changes in the spectral signature, greater in the low *CT* genotypes than in high *CT* ones, mainly in the *VIS*-*NIR* region. In fact, Weksler et al. (2020) report the association between changes in the transpiration rate and the spectral signature in pepper plants under different levels of potassium fertilization, evaluated every hour from 07:00 to 17:00 h; leaf spectral reflectance in the morning differed from those in the noon or afternoon, in the *VIS*-*NIR* region, as was observed in our results. In the case of the reflectance in the *NIR* region (700–1300 nm), it has been related to morphological characteristics of the leaf, such as cuticle, intercellular air space, the ratio between palisade mesophyll and spongy mesophyll (Peñuelas and Filella, 1998). In this sense, Willick et al. (2018) determined that, a drought tolerant wheat genotype differs from a susceptible in epidermal characteristics like, higher epicuticular wax density on the adaxial flag leaf surfaces and larger bulliform cells (Uddin and Marshall, 1988). We speculate that the evaluated genotypes in the present work may differ in these types of features, which may partly explain the differences in spectral responses, considering that they originated in different environments (breeding programs **Table 1**). These structural features have been associated with leaf water status and with several *SRIs* based on *NIR* wavelengths (El-Hendawy et al., 2019).

Significant peaks were found in the shortwave‐infrared range (*SWIR*, ~1,300 – 2,500 nm) that allow to differentiate the genotypes according to their *CT* (**Supplementary Figures S4A−E**, **S5**). The dynamics changes of reflectance peaks at 1,450 and 1,950 nm in the *UTALCA* experiment (daily and the diurnal progression analysis), could be explained by changes in tissue water content because that wavelength has been reported for estimating plant water status (Curcio and Petty, 1951; Palmer and Williams, 1974; Ihuoma and Madramootoo, 2019; Chandel et al., 2020). For example, El-Hendawy et al. (2019) identified wavelengths associated with changes in relative water content, developing a set of new *SRIs* for estimating leaf water status and grain yield of spring wheat grown under different irrigation regimes. Nevertheless, they also discuss about the low prediction capacity of *SRIs* when changes in the traits to be evaluated are slight to moderate (Lobos et al., 2014; Romero-Bravo et al., 2019).

It is well established that plants that maintain *gs* and *An*, under mild to moderate drought conditions (i.e., anisohydric stomatal control) have greater CO_2_ fixation, thus is usually associated with a greater tolerance to abiotic stress (Drew, 2006; Sade et al., 2009; Gallé et al., 2013; Rashid et al., 2018; Pawłowicz and Masajada, 2019). Nevertheless, the result of this work also suggests that the selection of higher *CT* (***H*** *_CT_-* ***H*** *_GY_*) material not always will turn into a low *GY* genotype.

New sources of genotypic variability for *GY* improvement are difficult to find, and this may compromise food security (Ray et al., 2013). If an isohydric genotype is selected in a high GY-based breeding program for tolerance to drought, the genotype might have the capability to mobilize a higher proportion of carbohydrates to the grain; as in the case of ***H*** *_CT_-* ***H*** *_GY_*, probably due to an improved harvest index (*HI*; Carmo-Silva et al., 2017). Nevertheless, according to this study, it appears that *GY* would be more associated with differences in the intrinsic water use efficiency between locations (*UTALCA vs*. *UIB*) than with the *CT* pattern; high *GY* genotypes showed a smaller distance between both *An*/*gs* regressions (**Supplementary Figures S3A, C**) compared to the low *GY* genotypes (**Supplementary Figures S3B, D**). A similar tendency of lower dispersion in spectral signature and gas exchange parameters (*gs* and *An*) was found in both high *CT* genotypes (isohydrics), which could be associated with their lower response to environmental fluctuation (**Supplementary Figures S4** , **S5**).

Finally, the present methodology, based on daily or diurnal progression analysis of spectral signature, appears to be a simple and consistent alternative to evaluate genotypic variability for a particular environmental condition (*G x E*), with the ability to discriminate between isohydric and anisohydric material; which is a trait used as selection criteria and for planning new crosses (Medina et al., 2019; Feng et al., 2020). This affirmation is reinforced when comparing the change in the daily and the diurnal progression of spectral signature (**Figures 2**, **4**). In particular, the selected wavelengths (380, 680, 1,450 and 1,950 nm, **Figures 6A–D**), that allow to differentiate the genotypes performance better than *Chl_f_* variables (*Fo*, *~Fo´*, *Fm* and *Fm´*) (**Figures 6E–H**). Also, the wavelengths showed higher and significant correlations with the chlorophyll fluorescence and gas exchange variables (**Supplementary Table 1**). Chlorophyll fluorescence and gas exchange parameters are widely used for genotype or cultivar selection under abiotic condition in different species (Flexas et al., 2000; Flexas et al., 2002; Estrada et al., 2015), however both measurements, at leaf level, are time consuming and therefore the number of measurements that can be performed is limited. In case of the *Chl_f_* measurements, methodologies have been developed to estimate sun-induced *Chl_f_* through spectral reflectance (proximally or remotely), to solve the time-consuming problem of measurements made at leaf level (Ni et al., 2019), increasing the number of genotypes evaluated in breeding programs (Azam et al., 2015; Bai et al., 2016; Song et al., 2018). Also, the two peaks of *Chl_f_*, centered at 685 nm and 740 nm (Corp et al., 2003; Meroni et al., 2009), have similar trends with *Chl_f_* variables, especially the one at 740 nm (**Supplementary Figure S6**). It is interesting to note the shape of *Fm* and *Fm´* of ***H*** *_CT_-* ***H*** *_GY_* across days, that could be an indicative of PSII photo-activity stability, through a constant capacity for harvesting and transfer of light energy in the mesophyll cells, which is reflected in *Fm* and *Fm´* behavior. On the contrary, gas exchange measurements still to be performed at leaf level, for what they are time consuming evaluation. In this sense, different approximation has been probed through modeling procedures using spectral reflectance information, to predict the stomatal conduce, the net CO_2_ assimilation and other variables (Garriga et al., 2017; Sexton et al., 2021). One of the advantages of the spectral reflectance measurements it’s that they are faster (seconds per leaf or at canopy level), and the protocols of measurements at the field are easy to implement, at the difference of gas exchange, *Chl_f_* and leaf or canopy temperature. In the particular case of the last type of measurement, different works have been developed to predict the canopy temperature by spectral reflectance information under abiotic stress conditions (Babar et al., 2006b; Kumagai et al., 2022); these arguments highlight that the methodology presented in this works, based on the interpretation of spectral signature patterns in a particular environment, is valuable to be used in the genotype selection with desirable characteristics, such as the transpiration regulation.

## 5 Conclusion

Spectral reflectance analysis showed the possibility of using the spectral signature to differentiate the contrasting responses of wheat genotypes to VPD fluctuations across days and hours. The low *CT* genotypes were more responsive to the environmental conditions, showing greater differences in their spectral signatures (mainly in the *VIS*-*NIR* region) in each environment, when comparing measurements between days and between hours, than the high *CT* genotypes. Four wavelengths, two in the *VIS* (380 and 680 nm) and two in the *SWIR* (1,450 and 1,950 nm) were identified as revealing the highest differences in the low *CT* genotypes. Spectral reflectance indices were also effective at evaluating the genotype sensitivity to stress conditions. Higher and significant correlations between *NDVI* and *PRI* with *VPD* and *RH* were obtained in the more sensible low *CT* genotypes. The spectral signature differences in low *CT* genotypes were associated with an anisohydric response to *WS* and *WS+T* due to higher *gs* and lower *T_l_*, while the higher *CT* showed an isohydric response.

This study highlights some perspectives in the use of spectral reflectance data for evaluating the plant response regarding to the changes in environmental conditions within a short period of time: i) the simple interpretation of the changes in the spectral signature by itself, due to environmental fluctuation, is a powerful tool for contrasting genotype performance (Lobos et al., 2019) related to isohydric or anisohydric responses, and ii) the wavelengths identified (380, 680, 1,450 and 1,950 nm) are interesting candidates to develop new spectral indices that allow evaluating genotypic sensibility to environmental changes.

The methodology used in this work reveals results that can be subsequently validated as a methodology using remote sensing tools in a large number of genotypes, to perform the first evaluations and segregation of genotypes in breeding programs.

## Data availability statement

The raw data supporting the conclusions of this article will be made available by the authors, without undue reservation.

## Author contributions

GL and FE contributed to the conception and design of the work. BC, CA-R, JG-T, JF, CD, FM-P, DC, IM, AM-E, MG, JA, and AP performed the analysis, and interpretation of data for the work. CD contributed to the experimental design, build of chambers, and measurements performed in UIB experiment in Spain. GL, JF, JA, AP, and FE collaborated to generate and validate the version to be published. All authors contributed to the article and approved the submitted version.
