# Supplementary material for: Exploring plant responses to abiotic stress by contrasting spectral signature changes
Source: Front Plant Sci. 2023 Jan 20;13:1026323. doi: 10.3389/fpls.2022.1026323 (PMC9910286; doi:10.3389/fpls.2022.1026323)
Supplement: Supplementary file 1 [file DataSheet_1.docx]

## Supplementary material


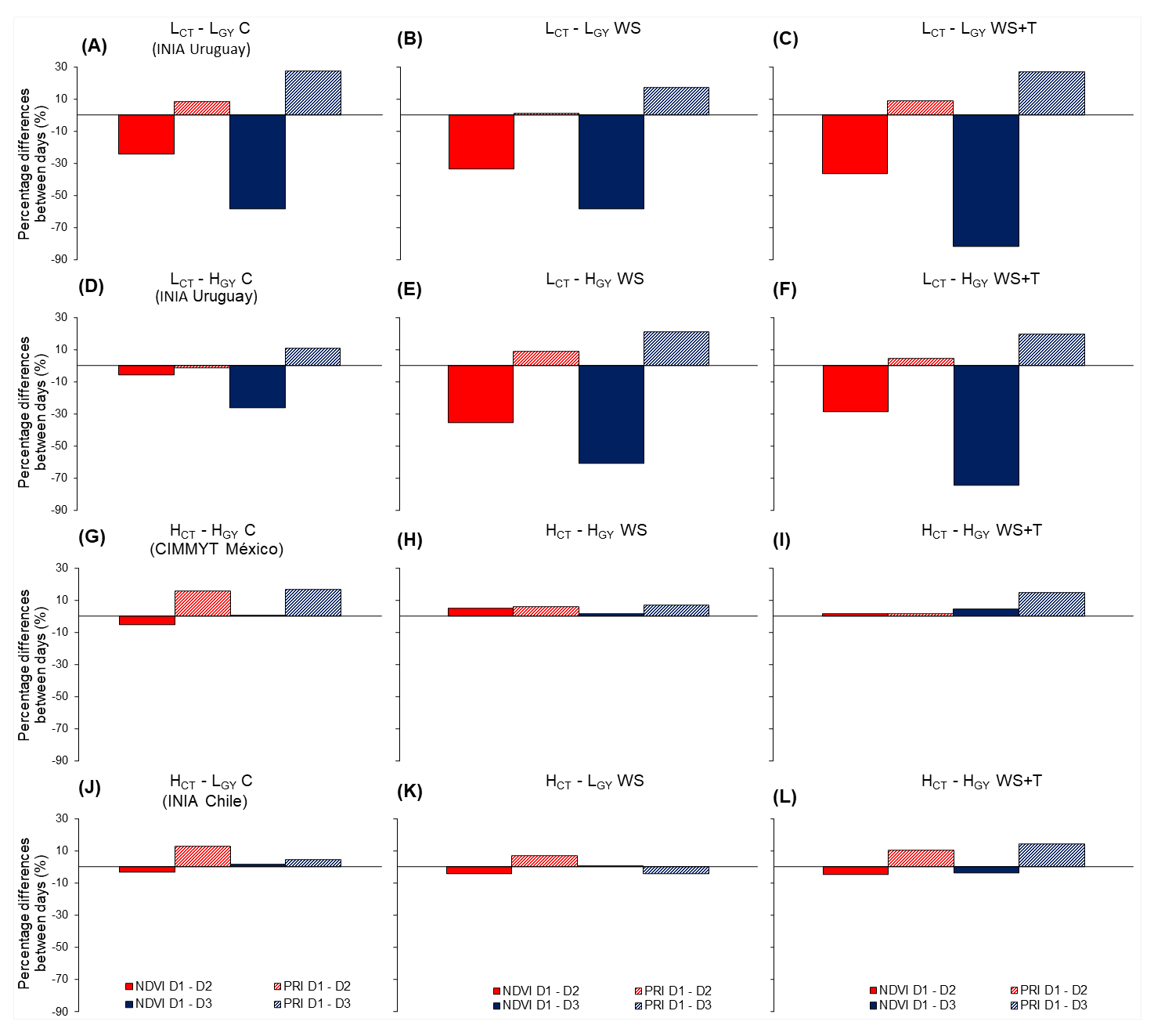


**Supplementary Figure S1.** Normalized difference vegetation index (*NDVI*; [R780 - R670] / [R780 + R670]) (solid bars) and Photochemical index (*PRI*; [R570 - R531 - R670] / [R570 + R531 + R670]) (dashed bars) of the percentage of change between days of assessment (*D1* to *D3: D1* - *D2* red bars, and *D1* - *D3* blue bars). Genotypes and treatments are indicated in Figure 1. Measurements were performed just before the plateau of the maximum daily atmospheric demand for water (~13:00 h); n = 9.


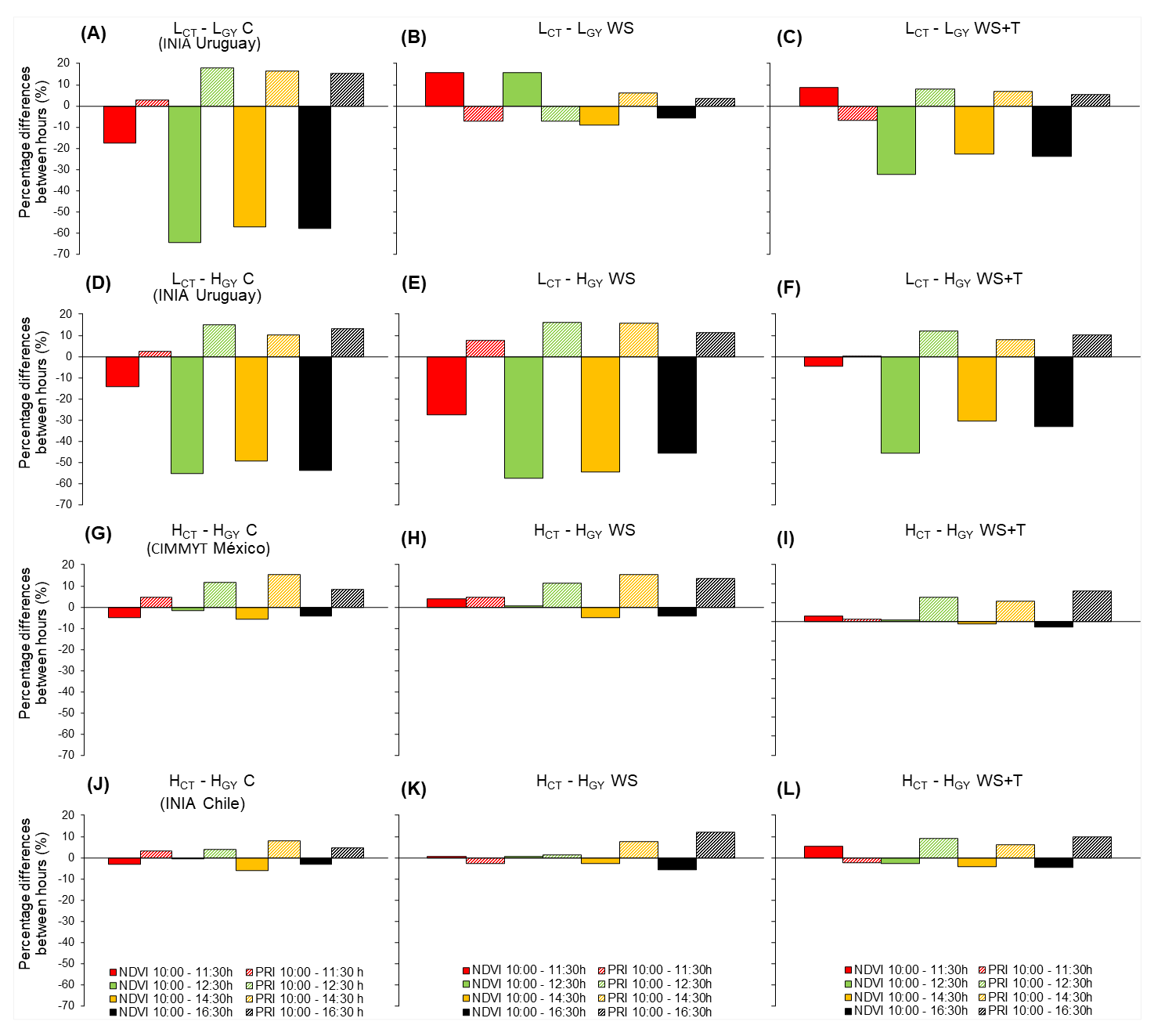


**Supplementary Figure S2.** Normalized difference vegetation index (*NDVI*; [R780 - R670] / [R780 + R670]) (solid bars) and Photochemical index (*PRI*; [R570 - R531 - R670] / [R570 + R531 + R670]) (dashed bars) of the percentage of change during the day (10:00/11:30 h red bar, 10:00/12:30 h green bar, 10:00/14:30 h yellow bar, and 10:00/16:30 h black bar). Genotypes and treatments are indicated in Figure 1. n = 9.


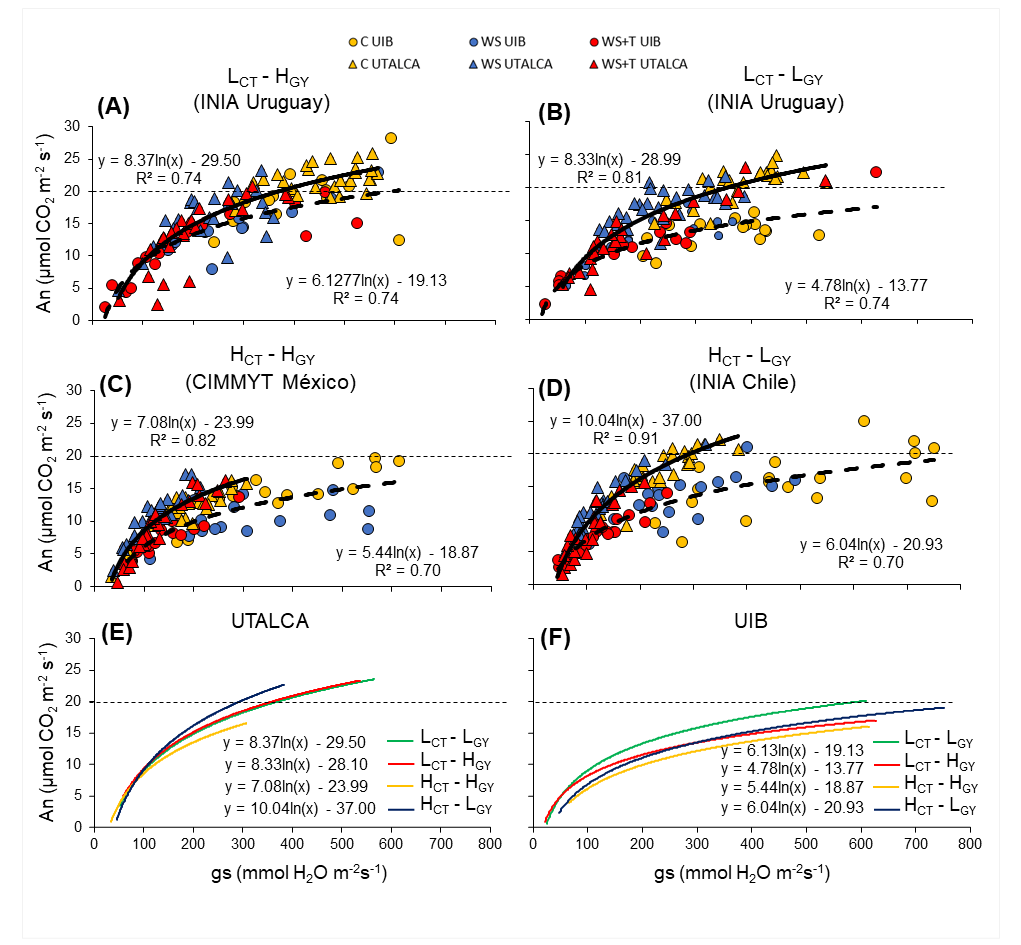


**Supplementary Figure S3.** Relationships between net CO_2_ assimilation (*An*) and stomatal conductance (*gs*) (intrinsic water use efficiency: *An*/*gs*), evaluated in the *UTALCA* experiment (triangles) during the 2018 season, and the *UIB* experiment (circles) during the 2019 season. Genotypes and treatments are indicated in Figure 1. Genotype comparisons at *UTALCA* (E) and *UIB* (F) are also shown. For reference purposes only, the horizontal black dashed line represents 20 µmol m^-2^ s^-1^.


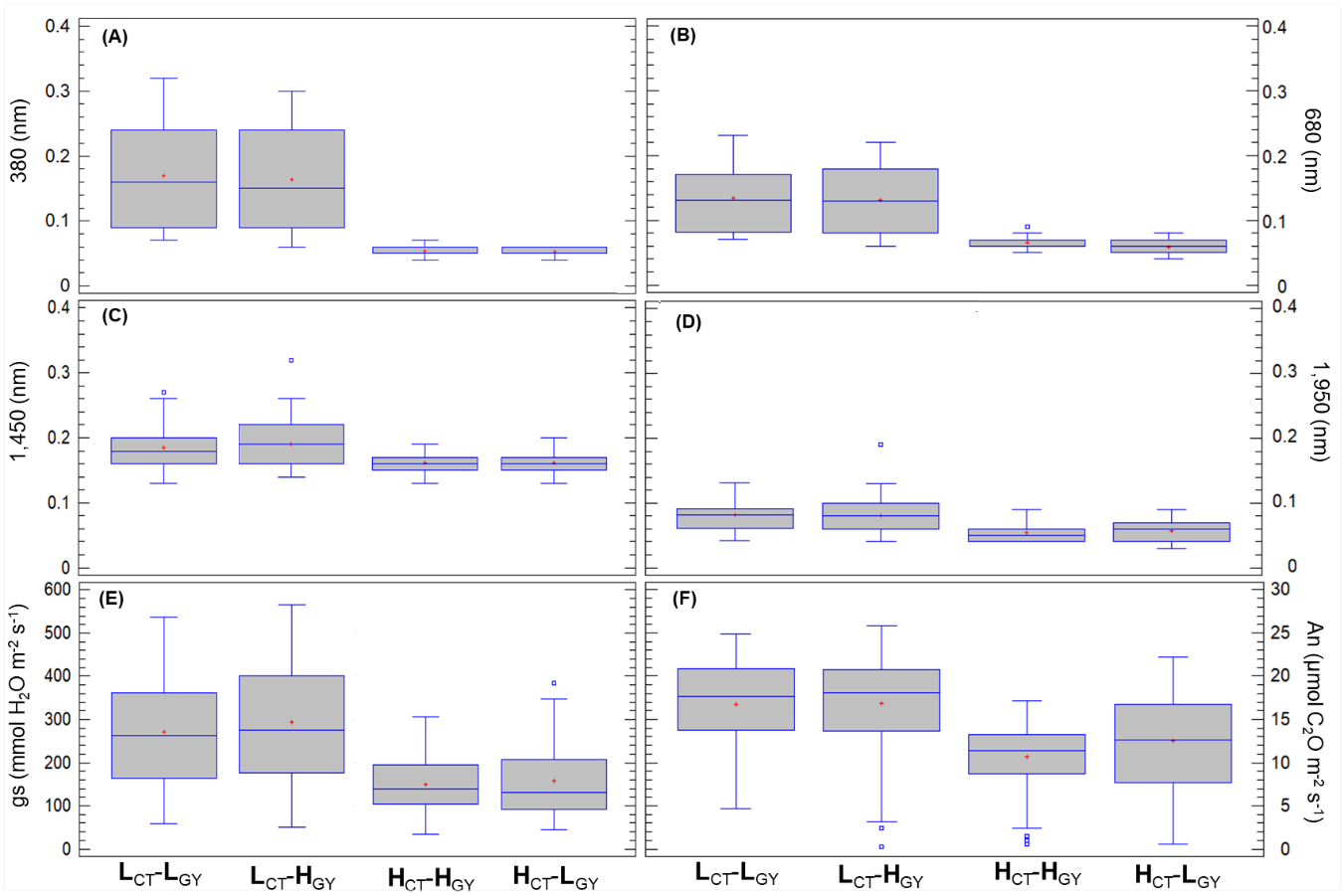


**Supplementary Figure S4.** Box and whisker plot of the wavelengths reflectance (380 nm: a, 680 nm: b, 1450 nm: c and 1950 nm: d), stomatal conductance (*gs*: e), and net CO_2_ assimilation (*An*: f), for each genotype, considering the data obtained at midday (*D1*, *D2*, and *D3*) in the three conditions (C, WS, and WS+T). The blue line in the box represents the median and the red cross the mean. n = 81.


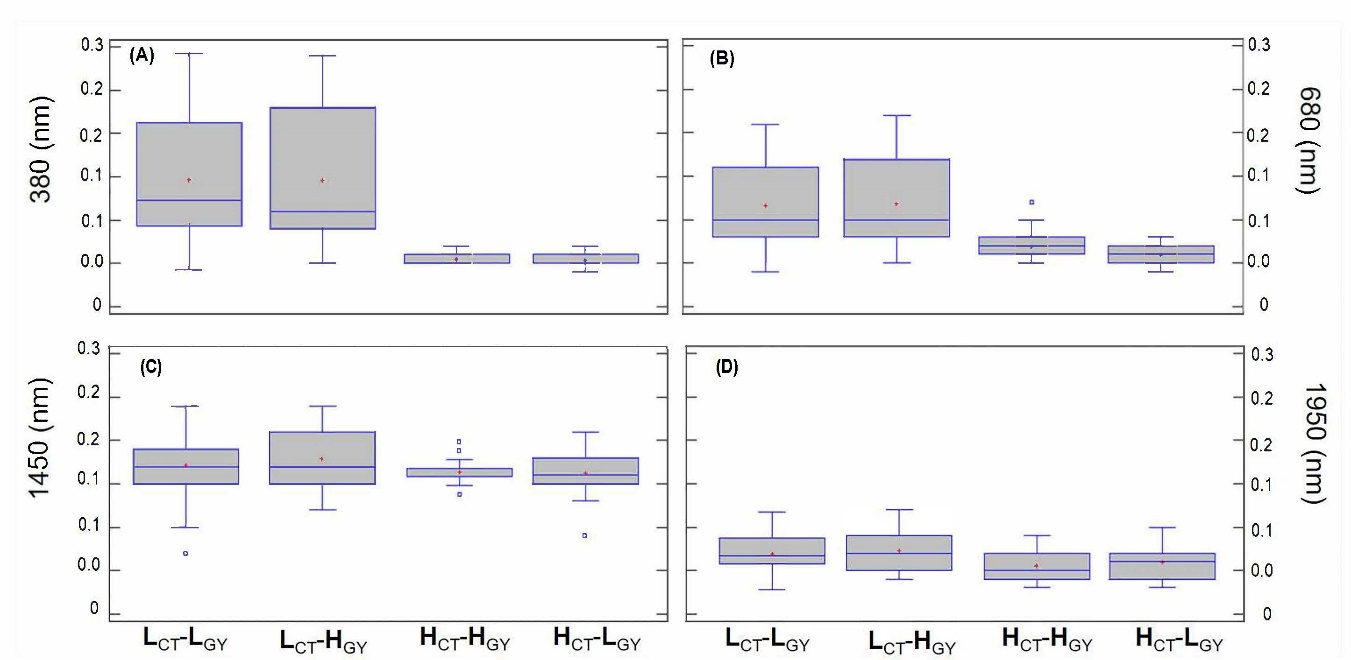


**Supplementary Figure S5.** Box and whisker plot of the wavelengths reflectance at 380 nm (a), 680 nm (b), 1450 nm (c) and 1950 nm (d), for each genotype, considering the hourly data (from 10:00 to 16:30 h) obtained on *D3* in the three conditions (*C*, *WS*, and *WS+T*). The blue line in the box represents the median and the red cross the mean. n = 135.


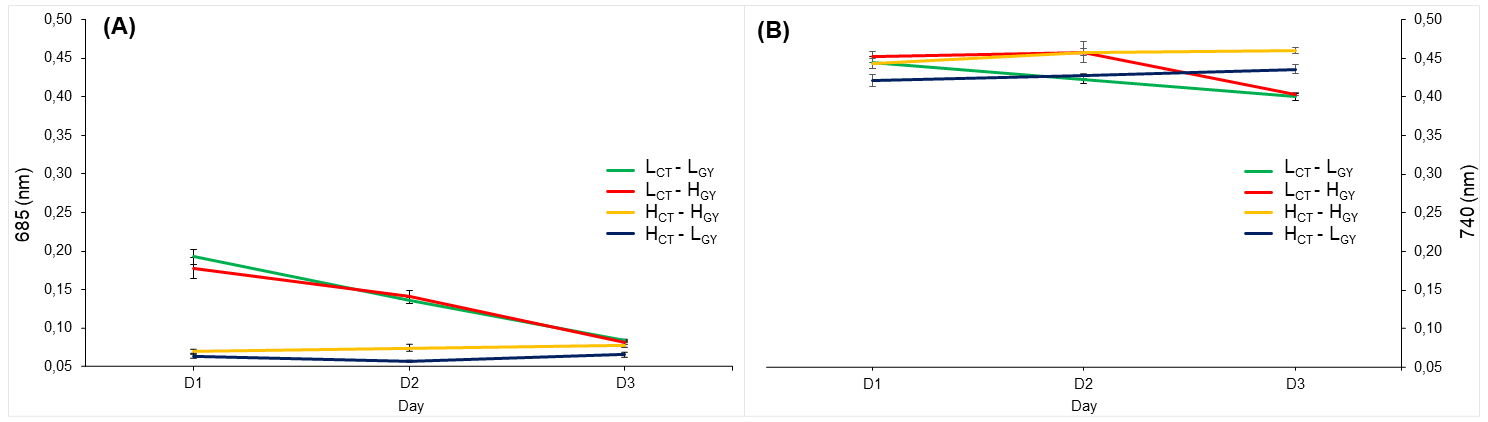


**Supplementary Figure S6.** Mean value of the reflectance at 650 nm (A) and 740 mm (B), in *UTALCA* experiment. n= 9.

**Supplementary table 1.** Pearson correlation analyses (*r*) of the relationships between physiological traits (leaf temperature; *T_l_*, stomatal conductance; *gs*, net CO_2_ assimilation; *An*, the minimum and the maximum yield of fluorescence under dark conditions, *Fo* and *Fm´,* respectively, the minimum calculated and the maximum yield of fluorescence under light conditions; ~*Fo´* and *Fm´,* respectively), with the reflectance at 380, 680, 1450 and 1950 wavelengths in the *UTALCA* experiment during the 2018 season, for the spring bread wheat genotypes on evaluated on three consecutive days (D1, D2 and D3), under control conditions (*C*), soil water stress (*WS*) and combined soil water and heat stress conditions (*WS+T*). Measurements were performed just before the plateau of the maximum daily atmospheric demand for water (~13:00 h). Values in bold correspond to correlations with p-values < 0.05; n = 12.

|  |  | D1 | | | | D2 | | | | D3 | | | |
| --- | --- | --- | --- | --- | --- | --- | --- | --- | --- | --- | --- | --- | --- |
| Treatment | Trait | 380 | 680 | 1450 | 1950 | 380 | 680 | 1450 | 1950 | 380 | 680 | 1450 | 1950 |
| C | T_l_ | **-0.8** | **-0.77** | -0.53 | -0.46 | 0.15 | 0.08 | -0.02 | 0.06 | **0.54** | 0.24 | -0.28 | -0.11 |
|  | gs | **0.65** | **0.6** | 0.37 | 0.27 | **0.8** | **0.84** | **0.74** | **0.81** | **0.59** | 0.46 | -0.01 | 0.21 |
|  | An | **0.58** | 0.52 | 0.23 | 0.2 | **0.68** | **0.67** | 0.51 | **0.7** | 0.45 | 0.35 | -0.13 | 0.03 |
|  | Fo | **0.78** | **0.77** | **0.59** | 0.55 | 0.2 | 0.23 | 0.44 | 0.24 | **0.81** | **0.62** | 0.41 | **0.58** |
|  | Fm | **0.81** | **0.76** | 0.48 | 0.44 | 0.19 | 0.21 | 0.13 | 0.29 | **0.88** | 0.47 | 0.29 | 0.54 |
|  | Fm´ | **0.75** | **0.71** | 0.43 | 0.39 | 0.39 | 0.43 | 0.39 | 0.44 | 0.55 | 0.05 | 0.35 | 0.45 |
|  | ~Fo´ | **0.77** | **0.74** | 0.52 | 0.48 | 0.22 | 0.27 | 0.47 | 0.25 | **0.68** | 0.33 | 0.49 | **0.57** |
| WS | T_l_ | **-0.91** | **-0.89** | **-0.86** | **-0.9** | -0.26 | -0.27 | -0.28 | -0.13 | **0.66** | **0.85** | **-0.73** | **-0.63** |
|  | gs | 0.55 | 0.53 | 0.44 | 0.55 | **0.88** | **0.8** | **0.74** | **0.73** | **0.77** | **0.6** | **-0.61** | -0.25 |
|  | An | **0.61** | **0.59** | 0.52 | **0.62** | **0.81** | **0.67** | **0.64** | **0.76** | **0.83** | **0.58** | **-0.62** | -0.33 |
|  | Fo | **0.91** | **0.91** | **0.8** | **0.87** | 0.31 | 0.42 | 0.46 | 0.3 | **0.61** | **0.71** | **-0.71** | **-0.59** |
|  | Fm | **0.84** | **0.83** | **0.74** | **0.82** | **0.77** | **0.56** | **0.74** | **0.86** | **0.71** | **0.66** | **-0.63** | -0.38 |
|  | Fm´ | **0.72** | **0.71** | **0.62** | **0.71** | 0.51 | 0.35 | 0.57 | 0.63 | 0.53 | 0.43 | -0.32 | -0.05 |
|  | ~Fo´ | **0.86** | **0.86** | **0.74** | **0.82** | 0.52 | 0.45 | 0.62 | 0.58 | 0.61 | 0.62 | **-0.57** | -0.39 |
| WS+T | T_l_ | **-0.93** | **-0.93** | **-0.88** | **-0.85** | 0.45 | 0.45 | 0.41 | 0.29 | 0.54 | 0.24 | -0.28 | -0.11 |
|  | gs | **0.73** | **0.73** | **0.65** | **0.59** | 0.52 | 0.55 | 0.46 | 0.54 | **0.59** | 0.46 | -0.01 | 0.21 |
|  | An | **0.8** | **0.79** | **0.68** | **0.63** | **0.58** | **0.6** | **0.57** | 0.55 | 0.45 | 0.35 | -0.13 | 0.03 |
|  | Fo | **0.88** | **0.88** | **0.89** | **0.87** | **0.8** | **0.83** | **0.77** | **0.86** | **0.81** | **0.62** | 0.41 | **0.58** |
|  | Fm | **0.79** | **0.81** | **0.83** | **0.85** | **0.75** | **0.69** | 0.57 | 0.57 | **0.88** | 0.47 | 0.29 | 0.54 |
|  | Fm´ | **0.84** | **0.85** | **0.88** | **0.85** | 0.3 | 0.24 | 0.32 | 0.16 | 0.55 | 0.05 | 0.35 | 0.45 |
|  | ~Fo´ | **0.87** | **0.87** | **0.89** | **0.86** | **0.72** | **0.72** | **0.76** | **0.72** | **0.68** | 0.33 | 0.49 | 0.57 |

**Supplementary table 2.** Mean values and variance analysis of the leaf temperature (*T_l_*) the stomatal conductance (*gs*) and the leaf net CO_2_ assimilation (*An*), the minimum and the maximum yield of fluorescence under dark conditions, *Fo* and *Fm´,* respectively, the minimum calculated and the maximum yield of fluorescence under light conditions; ~*Fo´* and *Fm´,* respectively), in four spring wheat genotypes growing under no stress (*C*), water stress (*WS*) and water stress combined with heat stress (*WS+T*) conditions, measured during three consecutive days (*D1*, *D2* and *D3*), at *UTALCA* and *UIB* experiments. Values are means (N=9). Means followed by the same letter are not significantly different (Tukey test, p < 0.05). Significance levels: *(P < 0.05), **(p < 0.01), ***(p < 0.001), n.s. (non-significant differences).

| **Experiment** | **Day** | **Trait** | ***L****_CT_-****L****_GY_* | ***L****_CT_-****H****_GY_* | ***H****_CT_-****H****_GY_* | ***H****_CT_-****L****_GY_* | **Treatment** | **Interaction** |
| --- | --- | --- | --- | --- | --- | --- | --- | --- |
| UTALCA | D1 |  |  |  |  |  |  |  |
|  |  | Tl (°C) | 26.5 | 24.2 | 32.4 | 31.8 | *** | n.s |
|  |  | gs (mmol H_2_O m^-2^s^-1^) | 225.6a | 273.7a | 120.0b | 146.9b | *** | *** |
|  |  | An (µmol CO_2_ m^-2^s^-1^) | 15.6a | 16.3a | 8.1b | 11.1b | *** | n.s |
|  |  | Fm | 3.3a | 3.2a | 2.6b | 2.7b | *** | *** |
|  |  | Fm´ | 1.5a | 1.5a | 1.2b | 1.1b | *** | n.s |
|  |  | Fo | 0.75a | 0.76a | 0.65b | 0.63b | *** | *** |
|  |  | ~Fo´ | 0.47b | 0.50b | 0.5a | 0.60a | *** | *** |
|  | D2 |  |  |  |  |  |  |  |
|  |  | Tl (°C) | 26.9 | 26.3 | 26.1 | 26.8 | *** | n.s |
|  |  | gs (mmol H_2_O m^-2^s^-1^) | 310.9a | 310.8a | 180.1b | 185.0b | *** | *** |
|  |  | An (µmol CO_2_ m^-2^s^-1^) | 18.0a | 16.7a | 12.0b | 14.0b | *** | n.s |
|  |  | Fm | 2.8b | 2.9a | 2.6c | 2.8b | *** | *** |
|  |  | Fm´ | 1.3bc | 1.4a | 1.2c | 1.3bc | *** | *** |
|  |  | Fo | 0.67b | 0.70a | 0.68ab | 0.64c | *** | n.s |
|  |  | ~Fo´ | 0.52b | 0.55a | 0.52b | 0.51b | *** | *** |
|  | D3 |  |  |  |  |  |  |  |
|  |  | Tl (°C) | 26.8ab | 27.4a | 26.0bc | 25.0c | *** | n.s |
|  |  | gs (mmol H_2_O m^-2^s^-1^) | 277.1a | 297.4a | 148.2b | 137.9b | *** | n.s |
|  |  | An (µmol CO_2_ m^-2^s^-1^) | 16.4a | 18.1a | 12.0b | 12.0b | *** | n.s |
|  |  | Fm | 3.0b | 3.1a | 2.6c | 2.7c | *** | *** |
|  |  | Fm´ | 1.3b | 1.5a | 1.2c | 1.2c | *** | *** |
|  |  | Fo | 0.69b | 0.73a | 0.67b | 0.64c | n.s | *** |
|  |  | ~Fo´ | 0.53b | 0.58a | 0.51c | 0.50c | *** | n.s |
| UIB | D1 |  |  |  |  |  |  |  |
|  |  | Tl (°C) | 34.1 | 33.3 | 34.2 | 34.1 | *** | *** |
|  |  | gs (mmol H_2_O m^-2^s^-1^) | 357.7 | 277.2 | 271.8 | 347.7 | *** | *** |
|  |  | An (µmol CO_2_ m^-2^s^-1^) | 13.0b | 17.2a | 12.0b | 14.5ab | *** | *** |
|  | D2 |  |  |  |  |  |  |  |
|  |  | Tl (°C) | 30.8b | 30.8b | 34.0a | 34.5a | *** | n.s |
|  |  | gs (mmol H_2_O m^-2^s^-1^) | 291.3 | 330.2 | 276.6 | 284.3 | *** | n.s |
|  |  | An (µmol CO_2_ m^-2^s^-1^) | 13.7ab | 16.1a | 10.0c | 11.9bc | *** | *** |
|  | D3 |  |  |  |  |  |  |  |
|  |  | Tl (°C) | 32.7 | 32.0 | 34.5 | 35.2 | *** | n.s |
|  |  | gs (mmol H_2_O m^-2^s^-1^) | 185.2 | 229.9 | 221.5 | 278.4 | *** | n.s |
|  |  | An (µmol CO_2_ m^-2^s^-1^) | 8.5b | 12.2a | 8.9b | 10.5ab | *** | n.s |
